# Diabetes, diabetes treatment, and mammographic density in Danish Diet, Cancer, and Health cohort

**DOI:** 10.1007/s10552-016-0829-z

**Published:** 2016-11-10

**Authors:** Karsten Buschard, Katrin Thomassen, Elsebeth Lynge, Ilse Vejborg, Anne Tjønneland, My von Euler-Chelpin, Zorana Jovanovic Andersen

**Affiliations:** 1 Bartholin Institute, Rigshospitalet, Blegdamsvej 9, 2100 Copenhagen, Denmark; 2 Department of Public Health, Center for Epidemiology and Screening, University of Copenhagen, Øster Farimagsgade 5, 1014 Copenhagen, Denmark; 3 Department of Radiology, Diagnostic Imaging Centre, Copenhagen University Hospital, Rigshospitalet, Blegdamsvej 9, 2100 Copenhagen, Denmark; 4 Danish Cancer Research Center, Danish Cancer Society, Strandboulevarden 49, Copenhagen, Denmark

**Keywords:** Diabetes, Mammographic density, Breast cancer, Insulin

## Abstract

**Purpose:**

We examined whether diabetes and diabetes treatment are associated with MD in a cohort study of Danish women above age of 50 years.

**Methods:**

Study cohort consisted of 5,644 women (4,500 postmenopausal) who participated in the Danish Diet, Cancer, and Health cohort (1993–1997) and subsequently attended mammographic screening in Copenhagen (1993–2001). We used MD assessed at the first screening after the cohort entry, defined as mixed/dense or fatty. Diabetes diagnoses and diabetes treatments (diet, insulin, or oral antidiabetic agents) were self-reported at the time of recruitment (1993–1997). The association between MD and diabetes was analyzed by logistic regression adjusted for potential confounders. Effect modification by menopausal status and body mass index (BMI) was performed by introducing an interaction term into the model and tested by Wald test.

**Results:**

Of 5,644 women with mean age of 56 years, 137 (2.4%) had diabetes and 3,180 (56.3%) had mixed/dense breasts. Having diabetes was significantly inversely associated with having mixed/dense breasts, in both, the crude model (odds ratio; 95% confidence interval: 0.33; 0.23–0.48), and after adjustment for adiposity and other risk factors (0.61; 0.40–0.92). Similar inverse associations were observed for 44 women who controlled diabetes by diet only and did not receive any medication (0.56; 0.27–1.14), and 62 who took oral antidiabetic agents only for diabetes (0.59; 0.32–1.09), while women taking insulin had increased odds of mixed/dense breasts (2.08; 0.68–6.35). There was no effect modification of these associations by menopausal status or BMI.

**Conclusions:**

Having diabetes controlled by diet or oral antidiabetic agents is associated with a decrease in MD, whereas taking insulin is associated with an increase in MD.

## Introduction

Numerous studies have demonstrated an association between type 2 diabetes and breast cancer [1–3]. A meta-analysis found a 27% increase in breast cancer risk in women with type 2 diabetes, which attenuated to 13% when adjusting for body mass index (BMI) [3]. Type 1 diabetes patients have no increased risk of breast cancer [4]. Exact biological mechanisms behind possible association between type 2 diabetes and breast cancer are unknown, and the relationship is complicated since diabetes and breast cancer share many risk factors including physical inactivity and obesity. Possible mechanisms include direct effects of hyperinsulinemia or the insulin-like growth factor (IGF) system on stimulating cell proliferation, as well as indirect effects mediated through altered levels of sex hormones [3]. Treatments which elevate circulating insulin levels in people with diabetes may increase cancer risk, and insulin analog glargine has been associated with higher risk of breast cancer than human insulin [5]. On the other hand, metformin, commonly prescribed used oral antidiabetic regimen, which increases insulin sensitivity and improves glycemic control, has been found to reduce breast cancer risk [6]. However, a large European study found no overall increase in breast cancer for type two diabetic patients, irrespective of type of treatment: sulfonylurea (hazard ratio (HR): 0.98), metformin (HR: 0.90), or insulin (HR: 1.07) [7].

One possible pathway from type 2 diabetes or diabetes treatment to breast cancer could be via an intermediary such as mammographic density (MD), one of the strongest risk factors for breast cancer [8]. MD refers to the amount of radiologically dense breast consisting of epithelial or stromal tissue that appears light on a mammogram [9]. Women with more than 75% density in the breast have a four to six times greater risk of breast cancer than women with little density, or fatty breasts [10]. Known determinants of MD include age at first birth, parity, age at menopause, hormone therapy (HT), all of which are estrogen-related, having a proliferative effect on fibroglandular tissue in the breast, increasing MD [10]. Use of chemopreventive agents, such as tamoxifen, can reduce MD [10]. Obesity, on the other hand, which increases the risk of postmenopausal breast cancer, likely via insulin as a mediator, decreases MD [10]. Only four studies examined the association between self-reported diabetes and MD [11–14], of which three reported slightly lower percent MD (PMD) in diabetic as compared to non-diabetic women [11–13], while a single study found statistically significant inverse association between diabetes and MD in premenopausal, but not in postmenopausal women [14]. No study to date had examined whether effect of diabetes on MD is differential by the type of treatment for diabetes.

Here we examined whether diabetes and diabetes treatment are associated with MD in a prospective cohort study of Danish women above age of 50 years.

## Methods

### Study population

The study population consists of 5,703 women above age 50 who participated in the Danish Diet, Cancer, and Health (DCH) cohort between 1993 and 1997 and subsequently attended the Copenhagen mammography screening program between 1993 and 2001.

### DCH cohort

Between 1993 and 1997, a total of 160,725 persons (72,729 women), 50–64 years of age, born in Denmark, living in Copenhagen or Aarhus (the two largest cities in Denmark), and free of cancer, were invited to participate in the DCH cohort study. A total of 57,053 people, of whom 29,875 were women (37% of invited women and 7% of entire Danish female population in this age group), accepted the invitation and participated in the study, answering a comprehensive questionnaire on diet, health, education, occupation, lifestyle, and reproductive factors. Height and weight were measured at the time of recruitment by a trained professional staff. Women were defined as premenopausal if they reported no HT use and at least 1 instance of menstruation ≤12 months before the time of recruitment, and postmenopausal otherwise. A detailed description of the DCH cohort has been published previously [15].

### Diabetes definition

Diabetes diagnosis (yes/no), age at diagnoses (years), and form of treatment for diabetes (diet regulated, insulin, or oral antidiabetic agents) were self-reported at the time of recruitment (1993-1997) in the DCH cohort. We defined diabetes as indicator (yes/no) of either having a diagnosis of diabetes or being treated for diabetes (diet, insulin or oral antidiabetic agents). Furthermore, we defined the three indicators of treatment for diabetes: diabetes controlled by diet only, insulin only, or oral antidiabetic agents only. We could not distinguish between type 1 and type 2 diabetes.

### Danish Copenhagen mammography register

The Copenhagen mammography screening program started in 1991 [16] and targeted approximately 40,000 women aged 50–69 years at the start of each biennial invitation round. We used data from the first five screening rounds between 1991 and 2001 [17]. Cases in which breast cancer was detected at the first screening were excluded from our final analytic data set, as these women lacked MD data.

### MD definition

One radiologist was in charge of the Copenhagen mammography screening program between 1991 and 2001 which took place at a single Copenhagen hospital, Rigshospitalet. All screens were taken by the radiographers or X-ray nurses, and were evaluated independently by two radiologists, who did not meet the attending women. A two-view mammography, craniocaudal and oblique, was performed at the initial screening. MD was dichotomized into fatty breast, equivalent to Breast Imaging Reporting and Data System (BI-RADS, Atlas, 2008) density code one and part of code two, and mixed/dense breast, equivalent to part of BI-RADS code two, and BI-RADS code three or four. Women with a negative screening test and fatty breasts were scheduled to have only an oblique view at their next screening, whereas women with a negative screening test and mixed/dense breasts were scheduled for another two-view mammogram. MD was not coded for positive screening mammograms. The dichotomous outcome for MD has been successfully utilized in earlier studies, showing the expected associations with breast cancer risk [17] and validated against BI-RADS density scores, with good agreement [18]. Using the personal identification number (CPR) of the Danish Civil Registration System [19], we linked the Copenhagen mammography register to the DCH cohort. We used MD assessed at the first screening after the cohort baseline (1993–1997).

### Statistical methods

We used logistic regression to investigate the association between diabetes and the three possible diabetes treatments (diet regulated/insulin/oral antidiabetic agents) and MD in separate models, and in four steps: crude model (Model 1); a model adjusted for age (Model 2); a Model 2 additionally adjusted for BMI (kg/m^2^) and waist circumference (cm) (Model 3), and Model 3 additionally adjusted for education (≤7 years/8–10 years/> 10 years), alcohol use (yes/no), alcohol intake (g/day), smoking (current/ever/never), physical activity in leisure time (yes/no), number of children, benign breast tumor (yes/no), and HT use (ever/never) (Model 4). We did not have data on chemopreventive agents in this cohort and did not include age at first birth and menopausal status in the model, as large number of women had missing data for these variables, 1,325 and 996, respectively. Analyses were stratified by menopausal status, overweight (BMI ≥ 25) and obesity (BMI ≥ 30). Effect modification of an association of MD with diabetes by menopausal status, overweight (BMI ≥ 25), and obesity (BMI ≥ 30) was analyzed by introducing an interaction term into the model and tested by Wald test. Logistic procedure in Stata 12.0 was used to conduct the analyses. Results are presented as odds ratios (ORs) with 95% confidence intervals (95% CI). We have run an additional model with diabetes defined as diabetes with onset after age 30 years, which could considered likely to be type two diabetics (excluding likely type 1 diabetes patients who typically are diagnosed before age 30 years).

## Results

Of 5,703 women in the study, we excluded 59 with missing data on one or more covariates, leaving 5,644 women for final analyses. Of these, 137 (2.4%) women had reported having diabetes at cohort baseline, 44 did not receive any medication and controlled diabetes by diet only, 62 took oral antidiabetic agents only, and 20 took insulin only, while 11 women answered that they used two regimens for treating diabetes, and were thus not included in the specific regimen analyses. The majority of women (56.3%) had mixed/dense breasts at their mammogram, which was taken at screening on average 1.1 years after the cohort baseline (93% had their mammogram within 2 years after baseline).

Mean age at baseline was 56 years, and 4,500 (79.7%) women were postmenopausal (Table 1). Mean BMI at baseline was 25.9 kg/m^2^, half (51.1%) of the women were overweight (BMI ≥ 25 kg/m^2^), and 16.7% were obese (BMI ≥ 30 kg/m^2^). Women with mixed/dense breasts were younger and had lower BMI than women with fatty breasts (Table 1). Mean age at diabetes diagnoses was 52.8 years, 59.5 years for women regulating diabetes by diet, 53.6 years for women taking oral antidiabetic agents, and 41.2 years for women taking insulin (Table 2). A total of 121 women received a diagnosis after age 30, which is considered to be most likely type 2 diabetes (Table 2), and in this group, age of onset of diabetes was 51.3 years for those taking insulin. Diabetic women taking insulin had lower BMI (24.0 kg/m^2^) than women regulating diabetes by diet (31.0 kg/m^2^) or taking oral antidiabetic agents (30.6 kg/m^2^). Of 16 who were diagnosed with diabetes before age 30 and had likely type 1 diabetes, seven had mixed/dense and five had fatty breasts (results not shown).

**Table 1:** Diabetes prevalence and characteristics for 5,644 women from Diet, Cancer, and Health cohort, by mammographic density

		Mammographic density	
	Total *n* = 5,644	Mixed/dense *n* = 3,180	Fatty *n* = 2,464
*Diabetes*			
Diabetes, *n* (%)	137 (2.4)	42 (1.3)	95 (3.9)
Diabetes controlled by diet only, *n* (%)	44 (0.8)	13 (0.4)	31 (1.3)
Diabetes treatment by oral antidiabetic agents only, *n* (%)	62 (1.1)	16 (0.5)	46 (1.9)
Diabetes treatment by insulin only, *n* (%)	20 (0.3)	15 (0.5)	5 (0.2)
*Cohort Participant Characteristics*			
Mean (SD) age (years)	56.3 (4.5)	55.4 (4.3)	57.3 (4.4)
Menopause, *n* (%)	4,500 (79.7)	2,444 (76.9)	2,056 (83.4)
Mean (SD) BMI (kg/m^2^)	25.9 (4.7)	24.6 (3.9)	27.6 (5.0)
Mean (SD) waist circumference (cm)	82.4 (11.8)	79.0 (10.1)	86.7 (12.4)
Overweight, *n* (%)	2,878 (51.0)	1,242 (39.1)	1,636 (66.4)
Obese (BMI > 30 kg/m^2^), *n* (%)	946 (16.8)	298 (9.4)	648 (26.3)
Short education (≤7 years), *n* (%)	2,023 (35.8)	988 (31.1)	1,035 (42.0)
Medium education (8–10 years), *n* (%)	2,757 (48.9)	1,601 (50.3)	1,156 (46.9)
Long education (>10 years), *n* (%)	864 (15.3)	591 (18.6)	273 (11.1)
Alcohol use, *n* (%)	5,454 (96.6)	3,082 (96.9)	2,372 (96.3)
Mean (SD) alcohol use in users (g/day)	13.8 (16.6)	14.8 (16.5)	12.6 (16.5)
Never smoked, *n* (%)	2,053 (36.5)	1,161 (36.6)	892 (36.4)
Previously smoked, *n* (%)	1,241 (22.1)	686 (21.6)	555 (22.6)
Current smoker, *n* (%)	2,330 (41.4)	1,324 (41.7)	1,006 (40.0)
Physically active, *n* (%)	2,696 (47.8)	1,571 (49.4)	1,125 (45.7)
Nulliparous, *n* (%)	835 (14.8)	559 (17.6)	276 (11.2)
Mean (SD) number of children^a^	2.2 (0.9)	2.0 (0.8)	2.3 (1.0)
Mean (SD) age at first birth (years)	22.6 (4.2)	22.8 (4.2)	22.3 (4.1)
Had benign breast tumor, *n* (%)	740 (13.1)	528 (16.6)	212 (8.6)
Ever used HT	2,705 (47.9)	1,633 (51.3)	1,072 (43.5)
Mean (SD) HT duration in ever users (years)	6.0 (6.0)	5.9 (5.9)	6.1 (6.1)

*SD* standard deviation, *BMI* body mass index, *HT* hormone therapy

^a^in parous women

**Table 2:** Adiposity and age at diagnoses distribution by diabetes treatment in 5,644 women from Diet, Cancer, and Health cohort

Total	Total *n* = 5,644	All diabetes *n* = 137	Diet *n* = 44	Oral antidiabetic agents *n* = 62	Insulin *n* = 20
Mean (SD) BMI (kg/m^2^)	25.9 (4.7)	30.1 (5.8)	31.0 (6.4)	30.6 (4.5)	24.0 (5.2)
Overweight, *n* (%)	2,878 (51.0)	110 (80.3)	38 (86.4)	54 (87.1)	6 (30.0)
Obese, *n* (%)	946 (16.8)	67 (48.9)	20 (45.4)	34 (54.8)	3 (15.0)
Mean (SD) age at diagnoses^a^	−	52.8 (14.5)	59.5 (14.1)	53.6 (13.1)	41.2 (19.5)

Diabetes above age 30	Total *n* = 5,640	All diabetes *n* = 121	Diet *n* = 36	Oral antidiabetic agents *n* = 56	Insulin *n* = 12
Mean (SD) BMI (kg/m^2^)	25.9 (4.7)	30.7 (5.5)	31.9 (6.4)	30.9 (4.5)	26.1 (5.8)
Overweight, *n* (%)	2,876 (51.0)	103 (85.1)	33 (91.7)	50 (89.3)	6 (50.0)
Obese, *n* (%)	946 (16.8)	64 (52.9)	18 (50.0)	32 (57.1)	3 (25.0)
Mean (SD) age at diagnoses^a^	−	56.6 (7.8)	59.6 (6.4)	57.0 (6.0)	51.3 (12.1)

Overweight = BMI > 25 kg/m^2^; Obese = BMI > 30 kg/m^2^

^a^for 133 women who have reported age at diabetes diagnoses

We found statistically significant inverse association between having diabetes and MD in a crude model (OR; 95% CI: 0.33; 0.23–0.48), which attenuated after adjustment for risk factors, especially adiposity, but remained statistically significant (0.61; 0.40–0.92) (Table 3). Similar inverse associations, although not statistically significant, were observed for women with diabetes controlled by diet only (0.56; 0.27–1.14), and for women taking oral antidiabetic agents only (0.59; 0.32–1.09) in the fully adjusted model. For women with diabetes taking insulin, we found a positive association with MD in all models, although statistically non-significant, due to small numbers (2.08; 0.68–6.35). Associations between diabetes and MD were slightly enhanced when limiting analyses to women with diabetes onset after age 30 (0.55; 0.35–0.87), mostly for women controlling diabetes by diet only (0.36; 0.15–0.86), while they remained unchanged for women taking oral antidiabetic agents (0.59; 0.31–1.13), and were slightly reduced for women taking insulin (2.01; 0.55–7.44), but there was no statically significant difference with estimates for all diabetes, regardless of age at onset (Table 3).

**Table 3:** Association between diabetes and MD among 5,644 women in Diet, Cancer, and Health cohort who participated in mammographic screening in Copenhagen

		MD		Model 1 Crude	Model 2 Age-adjusted	Model 3 Model 2 + adiposity^a^	Model 4 Fully adjusted^b^
		Mixed/dense	Fatty				
	*n*	*n*	*n*	OR (95% CI)	OR (95% CI)	OR (95% CI)	OR (95% CI)
No diabetes	5,507	3,138	2,369	1.00	1.00	1.00	1.00
All diabetes	137	42	95	0.33 (0.23–0.48)	0.34 (0.24–0.50)	0.62 (0.42–0.93)	0.61 (0.40–0.92)
Diabetes/diet only	44	13	31	0.32 (0.17–0.62)	0.33 (0.17–0.64)	0.62 (0.30–1.27)	0.56 (0.27–1.14)
Diabetes/oral antidiabetic agents only	62	16	46	0.27 (0.15–0.47)	0.27 (0.15–0.48)	0.56 (0.30–1.02)	0.59 (0.32–1.09)
Diabetes/insulin only	20	15	5	2.33 (0.85–6.43)	2.70 (0.96–7.54)	2.32 (0.78–6.90)	2.08 (0.68–6.36)
Diabetes above age 30^c^							
No diabetes	5,519	3,146	2,373	1.00	1.00	1.00	1.00
All diabetes	121	32	89	0.27 (0.18–0.41)	0.28 (0.18–0.42)	0.56 (0.36–0.86)	0.55 (0.35–0.87)
Diabetes/diet only	36	7	29	0.18 (0.08–0.42)	0.19 (0.08–0.45)	0.38 (0.15–0.92)	0.36 (0.15–0.86)
Diabetes/oral antidiabetic agents only	56	14	42	0.25 (0.14–0.47)	0.25 (0.14–0.47)	0.57 (0.30–1.08)	0.59 (0.31–1.13)
Diabetes/insulin only	12	8	4	1.55 (0.47–5.16)	1.85 (0.55–6.23)	2.12 (0.58–7.72)	2.01 (0.55–7.44)

*OR* odds ratio, *CI* confidence interval

^a^adjusted for age, body mass index (BMI) and waist circumference

^b^adjusted for age, BMI, waist circumference, menopausal status, education (<8 years, 8–10 years, >10 years), alcohol use (yes/no), alcohol intake (g/day), smoking (current/previous/never), number of children, benign breast tumor (yes/no), and HT use (ever/never)

^c^for 133 women who reported age at diabetes diagnoses

**Table 4:** Effect modification of the association^a^ between diabetes and MD by menopausal status and BMI, among 5,644 women in Diet, Cancer, and Health cohort who participated in mammographic screening in Copenhagen

	Mixed/dense *n*	Fatty *n*	OR (95% CI)	Mixed/dense *n*	Fatty *n*	OR (95% CI)	*p* value
	*Premenopausal* (*n* = 1,144)			*Postmenopausal* (*n* = 4,500)			
No diabetes	728	390	1.00	2,410	1,979	1.00	
All diabetes	8	18	0.51 (0.20–1.26)	34	77	0.64 (0.41–1.01)	0.57
Diabetes/diet	5	6	0.54 (0.14–2.13)	8	25	0.54 (0.23–1.27)	0.98
Diabetes/oral antidiabetic agents	3	9	0.52 (0.13–2.13)	13	37	0.60 (0.30–1.20)	0.80
Diabetes/insulin	2	1	2.40 (0.21–27.3)	13	4	2.14 (0.61–7.51)	0.92
	*Normal weight, BMI* < *25* (*n* = 2,766)			*Overweight, BMI* ≥ *25* (*n* = 2,878)			
No diabetes	1,921	818	1.00	1,217	1,551	1.00	
All diabetes	17	10	0.91 (0.39–2.11)	25	85	0.52 (0.32–0.84)	0.84
Diabetes/diet	5	1	1.81 (0.19–17.0)	8	30	0.43 (0.19–0.98)	0.24
Diabetes/oral antidiabetic agents	1	7	0.16 (0.02–1.39)	15	39	0.71 (0.37–1.35)	0.18
Diabetes/insulin	12	2	2.35 (0.48–11.6)	3	3	1.67 (0.31–9.11)	0.74
	*Not obese, BMI* < *30* (*n* = 4,698)			*Obese, BMI* ≥ *30* (n = 946)			
No diabetes	2,850	1,778	1.00	288	591	1.00	
All diabetes	32	38	0.69 (0.41–1.15)	10	57	0.43 (0.21–0.89)	0.44
Diabetes/diet	10	14	0.59 (0.24–1.40)	3	10	0.46 (0.13–1.67)	0.94
Diabetes/oral antidiabetic agents	9	19	0.51 (0.22–1.18)	7	27	0.61 (0.25–1.47)	0.63
Diabetes/insulin	15	2	3.72 (0.81–17.1)	0	3	NA	

*OR* odds ratio, *CI* confidence interval

^a^adjusted for age, body mass index (BMI) waist circumference, menopausal status, education (<8 years, 8–10 years, >10 years), alcohol use (yes/no), alcohol intake (g/day), smoking (current/previous/never), number of children, age at first birth, benign breast tumor (yes/no), and HT use (ever/never)

In stratified analyses, we found that there was no difference in association between diabetes and MD by menopausal status, or BMI (Table 4).

## Discussion

In this study, we present novel results of the differential association between diabetes and MD by diabetes treatment. Inverse associations between diabetes and MD were observed for women who controlled diabetes with diet or oral antidiabetic agents, while women taking insulin showed a positive association with having mixed/dense breasts, though statistically non-significant.

Our results generally agree with four studies on diabetes prevalence and MD, although differences in study design, study populations, and MD assessment preclude direct comparisons. The study by Tehranifar et al. [11] is a cross-sectional study from the New York Multiethnic Breast Cancer Project based on 124 pre- and 67 postmenopausal women with data on PMD, of whom 16 reported having type 2 diabetes. Mammograms were collected on the same date or 14 days after the interview. Diabetic women had slightly lower PMD than non-diabetic women, but the difference was not statistically significant [11]. Sellers et al. [12] utilized data from the Minnesota Breast Cancer Family Study Cohort to examine the association of PMD assessed shortly after interview in 2,530 women above age 40, where 161 reported diabetes, and found no statistically significant association, although mean PMD was slightly lower in diabetic women than in women without diabetes. Sanderson et al. [13] has in 476 black American women recruited at Meharry Medical College detected a lower percent breast density in 373 women with diabetes than in those without diabetes, but only in premenopausal women and without statistical significance, and no difference in postmenopausal women. Finally, Roubidoux et al. investigated the association between self-reported diabetes (*n* = 152) with MD available as BIRADS density scores among 144 pre- and 311 postmenopausal Southwestern Native American women, and found that diabetes was statistically significantly associated with lower BIRADS density in premenopausal women only, but found no association in postmenopausal women [14]. We found inverse, statistically significant associations between having diabetes and MD, in both pre- and postmenopausal women, although with slightly stronger associations in premenopausal women, in agreement with Sanderson et al. [13] and Roubidoux et al. [14]. Overall, evidence seems consistent that women with diabetes have less dense breasts than women without diabetes, in studies that control for BMI and adiposity. Diabetes reduces breast density, which is one of the strongest risk factors for breast cancer [10], but it, independently of breast density, increases the risk of breast cancer. The mechanisms by which type 2 diabetes increases the risk of breast cancer are not known, but several pathways are possible. Type 2 diabetes causes hyperglycemia, hyperinsulinemia, and increased inflammation, all of which may increase risk of breast cancer [1–3]. In addition, type 2 diabetes and breast cancer share many risk factors, including age, physical inactivity, overweight, and obesity, which may separately or together, increase risk of breast cancer in postmenopausal women with diabetes [1–3]. Overweight and obesity, for example, as type 2 diabetes, are associated with decrease in breast density [10], but increase in breast cancer risk in postmenopausal women.

We present the novel results that the association between diabetes and MD is differential with respect to type of treatment for diabetes. We found that women who take insulin have likely increased, whereas women taking oral antidiabetic agents or not taking any mediation have strongly decreased breast density, compared to women without diabetes. The exact biological mechanism behind these novel findings are not known, but some plausibility for the findings comes from existing evidence on associations between different diabetes treatment and breast cancer. Earlier studies have shown that insulin, a debated risk factor for breast cancer [4, 20], can stimulate cell proliferation in human breast cancer cell lines [21] and also in normal breast tissue [22, 23]. Thus, it is plausible that insulin can increase the amount of fibroglandular tissue in the breast, hence increasing MD [3, 20]. Several small studies examined association between fasting circulating insulin plasma levels and MD and found none, but have typically included healthy women without diabetes [24, 25]. Metformin, a biguanide, is the most commonly used oral medication for first-line treatment of diabetes. Metformin has multiple biological effects which can contribute to anticancer effects, including either direct antiproliferative effects or through indirect mechanisms, such as lowering of circulating insulin levels and improving glycemic control in diabetes patients [26]. In line with these physiological effects, metformin was found to lower postmenopausal breast cancer risk in some [6, 27] but not all studies [7]. Furthermore, metformin can reduce circulating androgen and estrogen levels [28]. Thus, it is plausible that metformin, by reducing levels of endogenous estrogen and cell proliferating insulin, can reduce MD. However, we found similar effect of decreased MD on both groups of women taking oral antidiabetic agents and regulating diabetes by diet only, precluding the conclusion that metformin alone can decrease MD, but rather suggesting that some other factor or characteristic common to both groups of diabetic women who did not take insulin contributes to lower MD.

This study benefited from having access to a large cohort of women with self-reported diabetes at recruitment in 1993–1999 as well as subsequent and independent collection of data on MD at breast cancer screening, facilitating the prospective design and limiting the possibility of recall or information bias. We had data and were able to adjust for all major diabetes and breast cancer risk factors and determinants of MD. Unlike any study before [11–14], we had objectively measured data on height and weight (BMI) and waist circumference, and were thus able to extensively adjust for adiposity, which is an important risk factor for diabetes and very important determinant for MD, as shown in Table 2. A major strength of this study is also the availability of information on diabetes treatment regiments, enabling us to examine the effect of diabetes regiments on MD for the first time. Furthermore, this is one of the largest studies to date on diabetes and MD, and perhaps the first study with enough power to detect statistically significant inverse associations between diabetes and MD, in contrast to earlier, smaller studies of typically few hundred patients [11–14]. Still, based on 137 diabetes cases, we still had limited power in the effect modification analyses. The main limitation is the possible misclassification of diabetes treatment, which is self-reported, as well as the small number of diabetes cases, limiting the power in analyses of diabetes treatment. Furthermore, we could not distinguish between type one and type 2 diabetes, but we found consistent results in a subset of women who most likely had type 2 diabetes (those with onset of diabetes above age 30 years). We also lacked the data on the specific type of insulin or oral antidiabetic agent regiments, although most of the patients in Denmark are prescribed metformin as oral diabetic agent. Recent report based on national data in Denmark between 2005 and 2012 showed that 81% of type 2 diabetes patients received metformin as their first antidiabetic medication, 13% started with sulfonylurea, and 6% with insulin [29]. We excluded women with positive outcome at the initial breast cancer screening, as they were not assigned MD, but instead referred to additional testing, by which we have likely excluded women with high MD, which is associated with breast cancer and low screening sensitivity. Another weakness is that DCH cohort participants are likely healthier than the general Danish population, implying some healthy worker effect, as it was shown that they are better educated and had higher income than non-participants [15]. Another limitation is that diabetes is self-reported, and likely underreported. However, self-reported diabetes prevalence in this cohort of 2.4% corresponds well to diabetes prevalence data for entire Denmark, based on Danish Diabetes register, which ranged from 1995 (first data in register) to 1997, in women, from 1.9 to 2.4%, [30].

In conclusion, we found that diabetic women had lower MD than women without diabetes, but that the association was differential by type of diabetes regimen. Having diabetes controlled by diet or oral antidiabetic agents seems to decrease, whereas taking insulin may increase MD. This information is important for women taking insulin and clinicians working with diabetes and breast cancer screening. Women with type 2 diabetes are at increased risk of breast cancer and have poorer prognosis of breast cancer [31], and as high risk group may have an added benefit from attending breast cancer screening and detecting cancer early. However, women with diabetes participate less in breast cancer screening than women from general population, and seem to miss out on the screening benefits [31]. Furthermore, increase in MD in women taking insulin may reduce the sensitivity of the screening in this group of diabetic women, as breast cancer screening performance decreases with increasing breast density [32]. Thus, diabetic women may benefit from better information on benefits of breast cancer screening, and the effect of their diabetes treatment regimen on breast density and related cancer screening performance, all of which may reduce breast cancer burden in this group of women. More research is needed to reproduce findings of this novel study.

## Abbreviations

MD: mammographic density
HT: hormone therapy
CPR: Danish personal identification number
DCH: Diet, Cancer, and Health cohort
BI-RADS: Breast Imaging Reporting and Data System
OR: odds ratio
CI: confidence interval
BMI: body mass index
